# Effects of Conventional Synthetic Disease-Modifying Antirheumatic Drugs on Response to Periodontal Treatment in Patients with Rheumatoid Arthritis

**DOI:** 10.1155/2018/1465402

**Published:** 2018-08-19

**Authors:** Gyu-Un Jung, Ji-Young Han, Kyung-Gyun Hwang, Chang-Joo Park, Panagiota G. Stathopoulou, Joseph P. Fiorellini

**Affiliations:** ^1^Department of Periodontology, Korea University Anam Hospital, Seoul, Republic of Korea; ^2^Division of Periodontology, Department of Dentistry, College of Medicine, Hanyang University, Seoul, Republic of Korea; ^3^Division of Oral and Maxillofacial Surgery, Department of Dentistry, College of Medicine, Hanyang University, Seoul, Republic of Korea; ^4^Department of Periodontics, University of Pennsylvania School of Dental Medicine, Philadelphia, PA, USA

## Abstract

Rheumatoid arthritis (RA) and periodontitis are common chronic inflammatory diseases and periodontitis is known to be more common and more severe in patients with RA. Based on a paucity of studies about the relationship between common conventional synthetic disease-modifying antirheumatic drugs (csDMARDs) and periodontitis, this prospective study aimed to evaluate the adjunctive effect of csDMARDs on response to nonsurgical periodontal treatment in patients with RA. Thirty-two patients with RA (RA group) and 32 systemically healthy patients (control group) with periodontitis were included in this study. The RA group patients were treated with csDMARDs, such as methotrexate, hydroxychloroquine, and sulfasalazine. Conventional nonsurgical periodontal treatment with scaling and root planing was performed in both groups. The extent and severity of periodontitis were evaluated by plaque index (PI), gingival index (GI), probing depth (PD), clinical attachment level (CAL), and bleeding on probing (BOP) at baseline and 4 weeks after periodontal treatment. There was no statistically significant difference of periodontal parameters between the RA and control groups at baseline. Four weeks after scaling and root planing, PD reduction, and CAL gain were higher in the RA group treated with csDMARDs compared to the control group, and the difference was statistically significant (*P* = 0.006 and 0.003, respectively). A* post hoc* analysis of the RA group showed no statistically significant difference on the response to nonsurgical periodontal treatment in multiple csDMARDs therapy and addition of NSAIDs and/or steroids to csDMARDs. In patients with RA, csDMARDs showed beneficial effect on periodontal clinical parameters following the nonsurgical periodontal treatment.

## 1. Introduction

Rheumatoid arthritis (RA) is a chronic, systemic immune-mediated inflammatory disease that may affect many tissues and organs, but primarily involves synovial joints [1]. Periodontitis is a chronic inflammatory process in which localized gingival inflammation due to bacteria destroys the alveolar bone and connective tissue that support the teeth [2]. In both diseases, the prevalence is low in young individuals and progressively increases with age [3–5]. It has been demonstrated that the prevalence of RA is highest especially in women older than 65 years [3].

The association between RA and periodontitis has long been studied and periodontitis is known to be more common and more severe in patients with RA [6–8]. Furthermore, both diseases have been suggested to share several pathogenic and pathologic characteristics [9, 10].* Porphyromonas gingivalis,* a known periodontal pathogen, has also been identified as playing a crucial role in the pathogenesis of RA [11]. In RA as well as in periodontitis, a chronic inflammatory reaction occurs in a confined space (the joint or gingival sulcus) and causes the destruction of adjacent bone [12]. Both diseases are believed to result from altered inflammatory and immunologic function, and similar cytokines and inflammatory mediators are present in synovial joints in patients with RA and in periodontal tissue in those with periodontitis [13–15].

Pharmacological treatment for RA includes agents targeted towards minimizing RA activity and reducing disease progression [16]. The drugs in current use are synthetic and biological disease-modifying antirheumatic drugs (DMARDs), nonsteroidal anti-inflammatory drugs (NSAIDs), and corticosteroids [17, 18]. The commonly used conventional synthetic DMARDs (csDMARDs) are methotrexate (MTX), leflunomide, hydroxychloroquine (HCQ), and sulfasalazine (SSZ) and they may be used as monotherapy or in combination. DMARDs can reduce or prevent joint damage and preserve joint integrity and function [19]. MTX has been the basic and first-line csDMARD for three decades and is still widely used. It is a folic acid antagonist that inhibits interleukin- (IL-) 1*β* and tumor necrosis factor- (TNF-) *α* production [20]. HCQ, an antimalarial drug that interferes with antigen presentation and immune activation by increasing the macrophage phagolysosomal pH, is usually prescribed in mild cases of RA [17]. SSZ contains multiple anti-inflammatory components and is cleaved in the colon into acetylsalicylic acid and sulfapyridin and its antirheumatic mechanism is thought to involve inhibition of proinflammatory transcription factors [17]. The biological DMARDs include TNF-*α* inhibitors, anti-B cell therapy, T-cell costimulation blockers, IL-6 inhibitors, IL-1 inhibitors, and protein kinase inhibitors [20, 21].

Pharmacological treatment of RA has been suggested to have positive effects on patients' periodontal status [16]. Several studies have indicated that biological DMARDs, sometimes classified as anticytokines, improve various measures of periodontal health [21–24]. Adalimumab, a TNF-*α* inhibitors, was recently shown to significantly decrease probing depth (PD) and bleeding on probing (BOP) in patients with RA [21]. Other common drugs for RA, such as NSAIDS and steroids, have been also suggested to affect the periodontal inflammatory status [22, 23], as determined by biomarkers of inflammation in the gingival crevicular fluid [24]. However, few authors have addressed the effects of the common csDMARDs therapy on periodontal tissue in a clinical setting [25]. Moreover, the effect of these agents on patients' response to periodontal therapy has not been studied.

In this study, our objective was to evaluate the effect of treatment with csDMARDs on the response to nonsurgical periodontal treatment in patients with RA.

## 2. Materials and Methods

### 2.1. Study Design

The study protocol was approved by the Institutional Review Board of Hanyang University Hospital (HUH 2011-R-17). The study was performed at the Division of Periodontology, Department of Dentistry, Hanyang University Hospital, from March 2011 to April 2013. All participants were informed of the aims and methods of the study, and written informed consent was obtained in advance. A total of 64 participants (14 men and 50 women, aged 32 to 79 years) were recruited; 32 participants with RA served as the test group (RA group) and 32 served as a systemically healthy control. The inclusion criteria for both groups were age of 30–80 years, ≥ 20 teeth present, and diagnosis of chronic periodontitis with at least 5 teeth with a clinical attachment level (CAL) of ≥ 4 mm. For the RA group, a confirmed diagnosis of RA was required by rheumatologist according to the 1987 revised criteria of the American Rheumatism Association [26]. In addition, as disease activity of RA is mostly assessed by determining the Disease Activity Score 28 (DAS28) including erythrocyte sedimentation rate (ESR) [12], inclusion criterion for the RA group was DAS 28-ESR > 3.2. The exclusion criteria for both groups were inability to perform oral hygiene, periodontal treatment within the last 3 months, use of antibiotic drugs within the last 1 month, use of anticoagulant medication, uncontrolled diabetes mellitus (DM), smoking ≥ 10 cigarettes/day, and pregnancy. All participants in the RA group had taken the csDMARDs and a combination of NSAIDs and/or steroids for management of RA. The csDMARDs used in this study included MTX, HCQ, and SSZ.

Full-mouth periodontal examination was performed by a calibrated periodontist (GUJ). The number of teeth present was recorded. The clinical periodontal parameters included the plaque index (PI) [27], gingival index (GI) [28], PD, CAL, and BOP. Briefly, the PI and GI were recorded for the Ramfjord teeth (the maxillary right first molar, maxillary left central incisor, maxillary left first premolar, mandibular left first molar, mandibular right central incisor, and mandibular right first premolar) and were expressed as scores of 0–3. The PD and CAL measurements were performed with a Williams probe at 6 sites per tooth (mesiobuccal, midbuccal, distobuccal, distolingual, midlingual, and mesiolingual) for all teeth except third molars. The BOP was recorded as present or absent within 30 seconds of probing at 6 sites per tooth for all teeth. All patients received full-mouth supra- and subgingival mechanical debridement in quadrant-wise basis at weekly interval by an examiner-blinded periodontist. The total time for quadrant-wise scaling/root planing required more than 4 hours. The clinical parameters were recorded again at reevaluation, 4 weeks after scaling/root planing. The measurements were recorded by the same periodontist blinded to the groups.

### 2.2. Statistical Analyses

Power analysis was performed with the statistical software (G*∗*Power, version 3.1.9, Kiel, Germany). A sample of 28 subjects per group would be required to show a significant difference in the periodontal measurements, with 80% power and 5% confidence [29]. Considering a possible attrition rate of 15% during the study period, the sample size of 32 subjects in each group was calculated. Clinical variables such as age, duration of RA, number of teeth, and periodontal parameters are presented as the mean ± SD. Differences between groups were compared by using the independent *t*-test (age, number of teeth, and sites with PD ≥ 4 mm) and *χ*^2^ test (gender, smoking, and DM) in SPSS (version 18.0, SPSS Inc., Chicago, IL).

The Kolmogorov Smirnov test and Levene's test were used to confirm the homogeneity of the normality and variances of the variables, respectively. Differences between the baseline and reevaluation in periodontal parameters were evaluated by independent *t* test. In the RA group, differences of periodontal parameters and DAS 28-ESR among subgroups divided by the prescribed csDMARDs and a combination of NSAIDs and/or steroids were analyzed using Kruskal-Wallis test. When any parameter was found to be statistically significant,* post hoc* analyses with Bonferroni adjustments were conducted to determine which pairwise comparisons were different. A *P* value of < 0.05 was considered indicative of statistical significance.

## 3. Results

The demographic data, number of teeth, smoking status, the presence of DM, and initial periodontal status (number of sites with PD ≥ 4 mm) of all participants are shown in Table 1. Thirty-two patients diagnosed with RA, 6 men and 26 women ranging in age from 32 to 76 years (60.4±10.0 years), were assigned to the RA group. The control group consisted of 8 men and 24 women aged 48 to 79 years (62.0±9.0 years). The groups were balanced in terms of gender, age, number of teeth, smoking status, the presence of DM, and initial periodontal status at the baseline. The mean duration of RA was 7.3±6.1 years and it could be assumed to represent the duration of therapy since the medication started immediately after the RA diagnosis.

The comparison of the clinical periodontal measurements between the RA group and the control group was shown in Table 2. There was no statistically significant difference between these groups for the PI, GI, PD, CAL, and BOP at baseline. Four weeks after scaling/root planing, all periodontal parameters are improved in both groups. Particularly, PD reduction was 0.6±0.4 mm for the control group and 0.8±0.4 mm for the RA group, and this difference was statistically significant (*P *= 0.006). CAL gain was 0.7±0.5 mm for the control group and 1.1±0.5 mm for the RA group, and this difference between groups was also statistically significant (*P* = 0.003).

All patients in the RA group took one or multiple of csDMARDs and some also received NSAIDs and/or steroids concurrently with csDMARDs. In* post hoc* analysis of RA group, a statistically significant difference was found in PD reduction between MTX only subgroup (0.5±0.2 mm) and MTX + HCQ + SSZ subgroup (1.1±0.5 mm) according to the different sets of csDMARDs (*P *= 0.002, Table 3). There was no significant difference of DAS 28-ESR and clinical periodontal measurements on the response to periodontal treatment when the subgroups were divided and compared according to the RA medication added to the csDMARDs (Table 4).

## 4. Discussion

RA and periodontitis are common, chronic inflammatory diseases with many pathologic and clinical similarities. RA afflicts approximately 0.5-1.0% of the population worldwide, and women are more frequently affected than men [1]. It can appear at any age, but its incidence is increasing with age [3–5]. Periodontitis, the most common cause of tooth loss in older people, also increases in prevalence and severity with age [30]. Individuals with RA are known to be at higher risk for periodontitis [7, 31]. Periodontitis has also been found to be more severe in patients with RA, who exhibit greater alveolar bone loss [32]. In addition, disability of the extremities and reduced manual dexterity caused by RA may impair oral hygiene and increase the risk for periodontitis and tooth loss [33]. Manifesting high levels of cytokines, such as IL-1*β*, IL-6, and TNF-*α*, periodontitis has an immunologic profile similar to that of RA and both diseases are associated with elevated concentrations of matrix metalloproteinases (MMPs) and low concentrations of tissue inhibitors of MMP (TIMPs) [13].

All RA patients in the present study received pharmacological treatment with csDMARDs alone or in a combination of NSAIDs and/or steroids in order to slow disease progression [34]. MTX, SSZ, and HCQ were the most commonly used DMARDs. Generally, treatment with MTX is initiated as soon as the clinical diagnosis of RA is confirmed [17, 19]. If a single csDMARD results in treatment failure or lack of efficacy, the clinician should consider adding a second csDMARD as a part of combination therapy or biological DMARDs [35]. Compared to the relatively new biological DMARDs, csDMARDs that are less expensive and exhibit less adverse effects remain as the mainstream pharmacological agents of choices for treatment of RA [19, 35]. In addition, all biological DMARDs have serious side effects related to secondary infections and potential risk for neoplasia, which may limit their use in long-term therapies in addition to their high cost [36].

RA patients with periodontitis on DMARDs have been found to exhibit lower local IL-1*β*, IL-4, and TNF-*α* levels than otherwise healthy patients with periodontitis [16], however, the study about the effect of DMARDs on clinical periodontal parameters is limited. The single or combined effects of DMARDs, NSAIDs, and steroids on the local anti-inflammatory cytokines and clinical periodontal parameters are also controversial [37–39]. With regard to biological DMARDs, it is reported that TNF-*α* inhibition alone limited an increase of CAL with no effect on inflammation [23]. Another study has found that there was no significant effect on the periodontal condition of RA patients in the absence of periodontal treatment [40]. In our study, since no statistically significant difference was found in any of the periodontal parameters between the RA group and the healthy control at baseline or prior to periodontal intervention, csDMARDs were thought to have no effects on the periodontal condition of RA patients as biological csDMARDs. Greater PD reduction and CAL gain were observed at 4 weeks after periodontal treatment in the RA group receiving csDMARDs. PD aFnon-surgucand CAL are the most reliable indicators of the extent and severity of periodontal disease; therefore, it means that csDMARDs could improve the periodontal condition of RA patients particularly when combined with the conventional periodontal therapy. It should be stated that some clinical periodontal parameters at baseline, for example, PD in Table 3 and CAL in Table 4, were statistically significantly different when the subgroups were divided and compared in the RA group. These differences at baseline seemed to induce the statistically significant PD reduction between MTX alone subgroup and MTX + HCQ + SSZ subgroup in* post hoc* comparison; however, they should be interpreted with caution due to a small sample size within each subgroup and a large variation of RA medication duration (7.3±6.1 years). Additionally, no synergistic effect of multiple csDMARDs or combinations of csDMARDs, NSAIDs, and/or steroids was observed on the response to periodontal treatment.

Compared to other studies [37–40], reevaluation was performed relatively earlier, at 4 weeks postoperatively since this study was designed to focus on the sole effects of csDMARDs on the response to nonsurgical periodontal treatment while minimizing the interfering effect of each patient's ability to perform proper oral hygiene in long-term follow-up study. Ideally, if another group of RA, who were treated only by nonsurgical periodontal therapy, were set, the adjunctive effects of DMARDs would have been clearly shown. We were not able to set this group of RA because csDMARDs must be inevitably prescribed to the RA patients to prevent the exacerbation of RA symptoms as soon as the RA diagnosis is confirmed [41]. The subgroup of HCQ + SSZ in the RA group was also not set because of our doctors' preference in the prescription.

The present study provides clinical evidence that the common csDMARDs might have an adjunctive effect on response to nonsurgical periodontal treatment in patients with RA. There was no statistically significant difference of DAS 28-ESR in the subgroups of RA; therefore, the beneficial effect of nonsurgical periodontal treatment for periodontitis might be more associated with the csDMARDs than the systemic amelioration of RA. Inflammation, however, is an intricate multiple step response triggered by various stimuli and csDMARDs can be involved in several stages in the inflammatory cascade. Further research is required to investigate the precise mechanism of csDMARDs in the pathway of periodontal inflammation and longer follow-up studies are necessary to determine the sustainability of this beneficial effects of the csDMARDs to nonsurgical periodontal treatment.

## 5. Conclusions

Within the limitations of this study, the following conclusions can be drawn. Four weeks after scaling and root planing, the PD reduction and CAL gain were statistically significantly greater in the RA patients on csDMARDs compared to the systemically healthy patients. There was no statistically significant difference in overall response to periodontal treatment based on the combination of multiple csDMARDs, NSAIDs, and steroids prescribed. The present study provides clinical evidence that csDMARDs may have an adjunctive effect on response to nonsurgical periodontal treatment in patients with RA.

## Figures and Tables

**Table 1:** Comparison of clinical characteristics between the healthy control and RA group.

	Control group	RA group	*P*
N	32	32	
Men/women	8/24	6/26	0.376
Age (years)	62.0 ± 9.0	60.4 ± 10.0	0.515
Number of teeth	26.3 ± 1.8	26.4 ± 1.5	0.709
Smokers	2	2	1.000
Diabetes mellitus	0	1	0.313
No. of sites with PD ≥ 4 mm (%)	9.8 ± 5.1	12.4 ± 11.2	0.371
RA duration (years)		7.3 ± 6.1	

Values are expressed as mean ± SD.

RA, rheumatoid arthritis.

^*∗*^ Independent *t*-test and *χ*^2^ test, respectively, *P *< 0.05.

**Table 2:** Response of periodontal parameters to non-surgical periodontal treatment compared between the healthy control and RA group.

		Control group	RA group	*P*
N		32	32	
PI	Pre	1.7 ± 0.6	1.9 ± 0.7	0.471
	Post	0.8 ± 0.4	1.1 ± 0.6	0.042
	Δ	-0.9 ± 0.7	-0.8 ± 0.7	0.354
GI	Pre	1.7 ± 0.5	1.6 ± 0.6	0.575
	Post	0.6 ± 0.4	0.7 ± 0.5	0.619
	Δ	-1.0 ± 0.7	-0.9 ± 0.6	0.400
PD	Pre	3.2 ± 0.4	3.0 ± 0.5	0.079
	Post	2.7 ± 0.3	2.2 ± 0.4	**0.001** **∗**
	Δ	-0.6 ± 0.4	-0.8 ± 0.4	**0.006** **∗**
CAL	Pre	4.0 ± 0.5	4.0 ± 0.6	0.907
	Post	3.3 ± 0.4	2.9 ± 0.6	**0.003** **∗**
	Δ	-0.7 ± 0.5	-1.1 ± 0.5	**0.003** *∗*
BOP	Pre	82.8 ± 24.1	78.4 ± 24.1	0.476
	Post	40.8 ± 25.8	39.4 ± 21.8	0.817
	Δ	-41.9 ± 35.7	-39.0 ± 26.5	0.711

Values are expressed as mean ± SD.

*Δ* is net change after nonsurgical periodontal treatment, calculated by Post-Pre.

BOP, bleeding on probing; CAL, clinical attachment level; GI, gingival index; PD, probing depth; PI, plaque index; RA, rheumatoid arthritis.

^*∗*^ Independent *t*-test, *P *< 0.05.

**Table 3:** Response of periodontal parameters to nonsurgical periodontal treatment among RA subgroup divided by csDMARDs used.

		MTX	HCQ	MTX	MTX	MTX	*P*
				+ HCQ	+ SSZ	+ HCQ	
						+ SSZ	
N		7	6	6	7	6	
PI	Pre	1.3 ± 0.6	2.1 ± 0.7	1.9 ± 0.3	2.0 ± 0.8	2.0 ± 0.6	0.104
	Post	0.8 ± 0.6	1.0 ± 0.4	1.1 ± 0.5	0.9 ± 0.5	1.6 ± 0.6	0.160
	*Δ*	-0.5 ± 0.5	-1.2 ± 0.6	-0.8 ± 0.6	-1.1 ± 0.6	-0.4 ± 0.7	0.127
GI	Pre	1.2 ± 0.7	1.7 ± 0.7	1.8 ± 0.4	1.4 ± 0.8	1.9 ± 0.5	0.378
	Post	0.7 ± 0.6	0.6 ± 0.6	0.8 ± 0.6	0.3 ± 0.2	1.0 ± 0.5	0.477
	*Δ*	-0.6 ± 0.3	-1.1 ± 0.7	-1.0 ± 0.7	-1.0 ± 0.8	-1.0 ± 0.7	0.606
PD	Pre	2.7 ± 0.4	3.0 ± 0.6	3.1 ± 0.1	2.7 ± 0.3	3.5 ± 0.5	**0.015** *∗*
	Post	2.2 ± 0.4	2.2 ± 0.4	2.3 ± 0.2	2.0 ± 0.1	2.4 ± 0.4	0.095
	*Δ*	-0.5 ± 0.2	-0.9 ± 0.2	-0.8 ± 0.2	-0.8 ± 0.3	-1.1 ± 0.5	**0.045** *∗*
CAL	Pre	3.9 ± 0.6	4.0 ± 0.5	4.2 ± 0.3	3.6 ± 0.8	4.1 ± 0.6	0.522
	Post	2.7 ± 0.5	3.0 ± 0.6	3.0 ± 0.6	2.8 ± 0.9	2.9 ± 0.4	0.793
	*Δ*	-1.2 ± 0.5	-0.9 ± 0.6	-1.2 ± 0.4	-0.8 ± 0.3	-1.2 ± 0.4	0.284
BOP	Pre	56.2 ± 26.3	71.4 ± 23.9	94.3 ± 8.8	89.8 ± 9.4	83.4 ± 25.4	0.058
	Post	37.5 ± 29.7	31.0 ± 15.5	44.4 ± 18.0	35.4 ± 8.8	47.9 ± 28.9	0.526
	*Δ*	-18.7 ± 21.5	-40.4 ± 16.7	-49.9 ± 19.0	-54.3 ± 14.8	-35.6 ± 39.9	0.094
DAS 28-ESR	Pre	4.2 ± 1.0	4.2 ± 0.9	4.0 ± 1.5	4.0 ± 1.6	4.3 ± 1.4	0.199
	Post	4.0 ± 1.2	4.1 ± 1.0	4.1 ± 1.1	4.0 ± 0.5	3.9 ± 1.1	0.254
	*Δ*	-0.1 ± 1.2	-0.1 ± 0.9	0.0 ± 1.2	0.0 ± 1.0	-0.3 ± 1.3	0.153

Values are expressed as mean ± SD.

*Δ* is net change after nonsurgical periodontal treatment, calculated by Post-Pre.

BOP, bleeding on probing; CAL, clinical attachment level; csDMARDs, conventional synthetic disease-modifying antirheumatic drugs; DAS 28-ESR, disease activity score 28-erythrocyte sedimentation rate; GI, gingival index; HCQ, hydroxychloroquine; MTX, methotrexate; NSAIDs, nonsteroidal anti-inflammatory drugs; PD, probing depth; PI, plaque index; RA, rheumatoid arthritis; SSZ, sulfasalazine.

*∗*Kruskal Wallis test, *P* < 0.05.

**Table 4:** Response of periodontal parameters to nonsurgical periodontal treatment among RA subgroups divided by the combination of csDMARDs, NSAIDs, and/or steroids.

		DMARDs	DMARDs	DMARDs	DMARDs	*P*
		alone	+ NSAIDs	+ steroids	+ NSAIDs	
					+ steroids	
N		8	4	7	13	
PI	Pre	1.9 ± 0.6	1.9 ± 0.9	1.7 ± 0.6	1.9 ± 0.7	0.751
	Post	1.0 ± 0.5	1.4 ± 0.8	1.0 ± 0.5	1.1 ± 0.6	0.800
	Δ	-0.9 ± 0.7	-0.6 ± 0.5	-0.7 ± 0.5	-0.8 ± 0.8	0.838
GI	Pre	1.8 ± 0.6	1.4 ± 0.8	1.3 ± 0.5	1.7 ± 0.7	0.370
	Post	0.7 ± 0.6	0.9 ± 0.5	0.7 ± 0.5	0.6 ± 0.6	0.663
	Δ	-1.1 ± 0.7	-0.5 ± 0.9	-0.6 ± 0.4	-1.1 ± 0.6	0.074
PD	Pre	3.0 ± 0.3	2.7 ± 0.3	2.9 ± 0.5	3.2 ± 0.6	0.195
	Post	2.2 ± 0.3	2.0 ± 0.1	2.3 ± 0.4	2.3 ± 0.4	0.568
	*Δ*	-0.8 ± 0.2	-0.7 ± 0.2	-0.6 ± 0.4	-0.9 ± 0.5	0.499
CAL	Pre	4.3 ± 0.3	3.3 ± 0.5	3.7 ± 0.6	4.1 ± 0.6	**0.036** *∗*
	Post	3.2 ± 0.5	2.2 ± 0.1	2.7 ± 0.7	3.0 ± 0.5	**0.016** *∗*
	Δ	-1.1 ± 0.4	-1.1 ± 0.5	-1.0 ± 0.5	-1.1 ± 0.5	0.861
BOP	Pre	84.3 ± 22.9	68.3 ± 23.8	69.1 ± 25.5	83.0 ± 24.3	0.167
	Post	36.1 ± 19.5	49.1 ± 12.9	34.5 ± 8.7	41.2 ± 29.4	0.609
	Δ	-48.2 ± 20.6	-38.9 ± 4.5	-34.6 ± 26.0	-41.8 ± 26.5	0.487
DAS 28-ESR	Pre	4.3 ± 0.9	4.4 ± 1.5	4.0 ± 1.6	4.2 ± 1.4	0.454
	Post	4.1 ± 1.0	4.1 ± 0.7	4.1 ± 1.2	3.9 ± 0.8	0.123
	Δ	-0.2 ± 1.0	-0.2 ± 1.0	0.1 ± 1.3	-0.2 ± 1.2	0.311

Values are expressed as mean ± SD.

Δ is net change after nonsurgical periodontal treatment, calculated by Post-Pre.

BOP, bleeding on probing; CAL, clinical attachment level; csDMARDs, conventional synthetic disease-modifying antirheumatic drugs; DAS 28-ESR, disease activity score 28-erythrocyte sedimentation rate; GI, gingival index; NSAIDs, nonsteroidal anti-inflammatory drugs; PD, probing depth; PI, plaque index; RA, rheumatoid arthritis.

*∗*Kruskal Wallis test, *P* < 0.05.

## Data Availability

The data used to support the findings of this study are available from the corresponding author upon request.
